# Severe Unilateral Microtia with Aural Atresia, Hair White Patch, Stereotypes in a Young Boy with De novo 16p13.11 Deletion: Reasons for a New Genotype–Phenotype Correlation

**DOI:** 10.1055/s-0043-1777362

**Published:** 2023-12-04

**Authors:** Piero Pavone, Xena Giada Pappalardo, Claudia Parano, Enrico Parano, Antonio Corsello, Martino Ruggieri, Giovanni Cacciaguerra, Raffaele Falsaperla

**Affiliations:** 1 Section of Paediatrics and Child Neuropsychiatry, Department of Clinical and Experimental Medicine, University of Catania, Catania, Italy; 2 Unit of Catania, Institute for Biomedical Research and Innovation, National Council of Research, Catania, Italy; 3 Department of General Surgery and Medical-Surgical Specialties, University of Catania, Catania, Italy; 4 Neonatal Intensive Care Unit, Department of Sciences for Health Promotion, Maternal Infant Care, Internal Medicine and Medical Specialties “G. D'Alessandro,” University Hospital “P. Giaccone,” Palermo, Italy; 5 Section of Paediatrics and Child Neuropsychiatry, Unit of Rare Diseases of the Nervous System in Childhood, Department of Clinical and Experimental Medicine, University of Catania, Catania, Italy; 6 Neonatal Intensive Care Unit, AUO Policlinico “Rodolico-San Marco,” University of Catania, Catania, Italy; 7 Acute End Emergency Pediatric Unit, Department of General Pediatrics, AUO Policlinico “Rodolico-San Marco,” University of Catania, Catania, Italy

**Keywords:** case report, microtia, 16p13.11 deletion, genotype–phenotype correlation

## Abstract

**Background** Microtia is an uncommon congenital malformation ranging from mild anatomic structural abnormalities to partial or complete absence of the ear leading to hearing impairment. Congenital microtia may present as a single malformation (isolated microtia) or sometimes associated with other congenital anomalies involving various organs. Microtia has been classified in three degrees according to the complexity of the auricular malformation and to anotia referred to the total absence of the ear. Genetic role in causing auricular malformation has been widely demonstrated, and genotype–phenotype correlation has been reported in cases of syndromic microtia.

**Case Presentation** We report here a young patient with a third degree of scale classification and aural atresia. The patient showed unspecific facial dysmorphism, speech delay, precocious teething, hair white patch, and stereotypic anomalous movements. Genetic analysis displayed a de novo 16p13.11 deletion.

**Conclusion** Microtia with aural atresia is an uncommon and severe birth defect, which affects functional and esthetic aspects, often associated with other malformations. As traumatic this disorder may be for the parents, the microtia and aural atresia are treatable, thanks to the improving and evolving surgical techniques. Based on the genetic analysis and the clinical features observed in the present case, a genotype–phenotype correlation has been proposed.

## Introduction

Microtia including anotia is an uncommon but often severe congenital auricular malformation. It is a birth defect with a wide clinical expressivity ranging from mild anatomic structural abnormalities to partial or complete absence of the ear and hearing impairments. 1 Since there is often a correlation between the degree of auricular deformity and the middle ear involvement, the cases with severe microtia are frequently associated with aural atresia, a condition in which both the middle ear and external auditory canal fail to form. 2 The prevalence of microtia estimated between 0.84 and 17.4 per 10,000 birth is also significantly influenced by the geographic variability. 3 4 5 Harris et al reported on a prevalence of 0.23 for anotia and of 1.55 for microtia per 10,000 births, respectively. 6

The auricular malformation most commonly occurs in males and in a large series of 156 patients, male prevalence was found in 56% of cases and females in 44%. 1 The anomaly is often unilateral with the right side more frequently affected. 1 5 Microtia may be acquired or congenital, the last one presenting as a single malformation (isolated microtia), often associated with wide and different types of others congenital anomalies, or to be a sign of several rare syndromes (syndromic microtia). Microtia has been associated with malformations involving various organs including facial, cardiac, renal, ocular (micro- and anophthalmia), cerebral (holoprosencephaly), and skeletal. 1 7 8 Examples of syndromic microtia are represented by the syndromes of Goldenhar, Treacher Collins, Nager, Crouzon, Pierre Robin among the others, and in the group of spectrum disorders, the oculoauricolovertebral, branchio-oto-renal, and others. 5 9 10 Although relevant progresses have been reached, particularly in the genetics of microtia and associated syndromes, specific etiopathogenetic mechanisms remains to be established. The anomaly is thought to be multifactorial with a relevant causal role linked to variable combination of environmental, genetic, and epigenetic factors. Mendelian inheritance pattern has been reported more frequently in syndromic and familial cases of microtia, whereas polygenic factors has been shown to prevail in sporadic cases. Genetic role in the etiopathogenesis of the auricular malformation has been widely demonstrated by the concordance of the clinical features observed in monozygotic twins and by the occurrence of autosomal recessive or dominant inheritance observed in some of the families affected. 5 10 One set of identical twins demonstrated severe microtia in one twin yet only a minimal anomaly in the other. 1 Some genotype–phenotype correlations have been shown in several cases of syndromic microtia as follows: the genes *SIX5* and *EYA1* have been detected in the branchio-oto-renal syndrome; the genes *TCOF1* , *POL1RC* , and *POL1RD* in Treacher Collins' syndrome; *HMX1* and *OTX2* in the oculoauriculovertebral spectrum; *SF3B4* in Nager's syndrome; *SALL1* in Townes–Brock's syndrome, and *HOXA2* in microtia, hearing impairment, and cleft palate. 5 11 12 13 14 15 The screening of the *BMP5* locus have shown a missense mutation in four cases 13 and five genomic variants in *GSC* , *HOXA2* , *PRKRA* genes have been reported in a group of 106 patients with congenital microtia. 16 In a five-generation kindred study in patients affected by microtia, Quiat et al 17 identified a missense variant in *FOX13* (p.Arg236Trp) gene as causal factor of the anomaly. Moreover, deletions in 6q13–q15, in 12p13.33 involving the *WNT5B* gene, in 22q11.2 involving *TBX1* gene, 20q13.33 have been also associated with syndromes including congenital ear malformation. 10 18 19 Veltman et al have also indicated the 18q22.3–18q23 as a candidate chromosomal region for the congenital aural atresia. 20 The 16p13.11 microdeletion syndrome (ORPHA:261236) is characterized by developmental delay, microcephaly, epilepsy, short stature, facial dysmorphism, and behavioral problems. 21 Genetic findings disclosed that some genes mapped in the 16p13.11 locus could be involved in a wide spectrum of multiple congenital anomalies such as facial dysmorphisms and microtia. 15 21 22 23 24

Here, we reported on a clinical case affected by a de novo 16p13.11 microdeletion and microtia attempting to provide some insights to further define the role of deleted genes on ear malformation.

## Case Presentation

### Clinical History

**Fig. 1:**
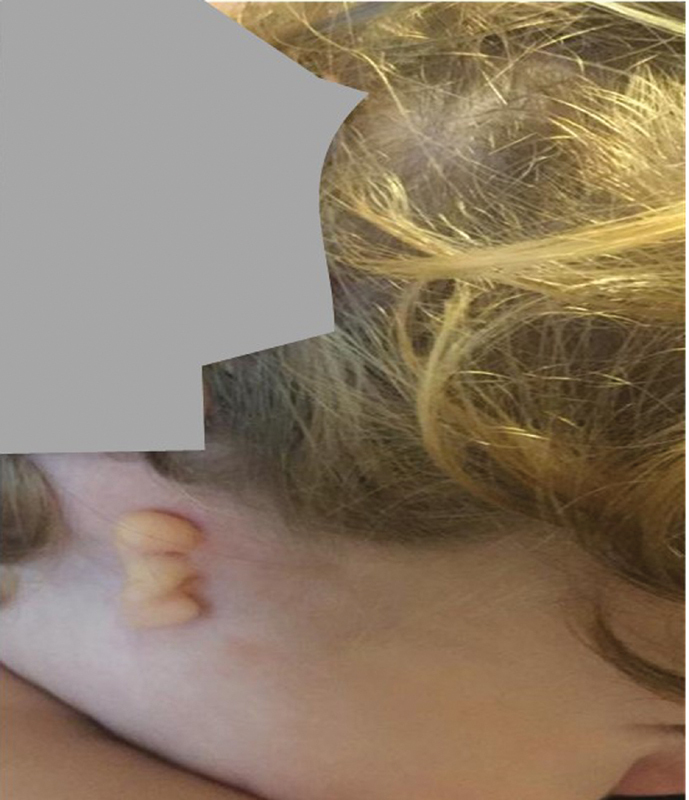
The auricular malformation with third degree of scale of Hunter's classification and white patch.

A 19-month-old boy is the first born of unrelated Italian parents. Family history is irrelevant and both parents are healthy with a good level of instruction, and no record of past or present relevant clinical disturbances. The mother have had formerly a miscarriage very early in the pregnancy. At the time of conception, the father was 29 years old and the mother 27 years old. At 6 months of gestation, the mother complained of febrile infection lasted 3 days and treated with antibiotic. No anomalies were reported at the intrauterine ultrasound. The child was born at 37 weeks of gestation by urgent cesarean section due to sudden placental abruption. The birth weight was 3.420 kg, height 48 cm, and occipitofrontal circumference (OFC) 35 cm (all within normal range). Apgar score was 9 at 1 minute and 10 at 5 minutes, SaO _2_ 98% and normal hearth rate. The right ear malformation was immediately noted. At the second day of life, the newborn showed poor reactivity with poor suction and bradycardia heart rate 80 to 100 beats/min. At this age, electrocardiogram (ECG) and echocardiogram displayed cardiac normal structure and function. At the fourth day of life, the neonatal icterus with value of total bilirubin of 20.8 mg/dL was found and treated with phototherapy. He was discharged in good condition with a diagnosis of right ear malformation and neonatal icterus. During the following months, the child showed a normal ponderal and statural growth, marked speech delay, and precocious teething. Several examinations were performed: otorhinolaryngological and ophthalmological examinations were normal, as well as ECG and echocardiogram. Brain magnetic resonance imaging (MRI) performed at the age of 7 months showed no alterations as regards cerebral and cerebellar parenchyma. Facial skull MR showed external auditory canal atresia, whereas the structures of the inner ear (cochlea and semicircular canals) and status of acoustic nerve were normal. No radiographic lesion in the right mandibular was noted. At the recent observation, at 19 months old, the child shows to be alert and in good condition, his weight is 13.3 kg (75 percentile, pc), height is 83 cm (50 pc), and OFC 49 cm (75 pc). At physical examination, he shows unspecific facial dysmorphism with epicanthus, poor eyelids, short nose, and rounded nasal tip. His hairs are blond with white patch (albinism type). The left ear is normal. The right auricle appears abortive, malformed, small, without any recognizable anatomic component. It appears as a linear, fleshy, and cartilaginous remnants sized 3 cm × 1 cm. The anomaly appears to be distinct in three sectors by two deep introflexions, and it designs the embryological origin from branchial arch: the helix, helical root, and tragus. Moreover, from the inferior part of the malformed auricle, at palpation, a subcutaneous thin, linear subcutaneous thickening of fibrotic consistence ∼5 cm long with a small furrow in the middle of its course and following the mandibular line is detected ( Fig. 1 ). Moreover, our proband showed the third degree of the microtia showed the most severe degree of the microtia (third degree) based on the Hunter's classification. 24 Heart, thorax, abdomen, and internal organs are normal. No kidneys' anomalies are found with abdominal ultrasound. At the neurologic examination, the cranial nerves are intact including facial nerve, muscle tonus and deep patellar reflexes are normally present, and had normal gait. He shows marked speech delay, hyperactivity, and stereotypies consisting in repetitive crossing and uncrossing upper legs movements. He is a well affective and socialized boy. Present laboratory testing provides normal results, including plasma and urinary amino acids, organic acids, thyroid and celiac markers, and total cholesterol. Fundus oculi, abdominal and scrotal ultrasound, and left otoacoustic emissions are normal.

## Materials and Methods

### Molecular Genetic Findings

Genomic DNA was isolated from the peripheral blood of the proband and parents to analyze structural anomalies using Human Genome CGH Microarray kit 8 × 60K performed according to the manufacturer's recommendations (Agilent Technologies, Santa Clara, California, United States). Data analysis was done using Cytogenomics v.4.0.3 software (genomic assembly UCSC hg 19) with quality score (derivative log ratio spread) of <0.25. The following analysis parameters were applied: five consecutive probes, automated decision-making-2 algorithm, algorithm threshold 6.0, and resolution of 100 to 200 Kb. The array comparative genome hybridization revealed in the proband the following rearrangement: arr[hg19]16p13.11(15.048.751–16.249.607)x1. The rearrangement consists of a 1.2 Mb deletion of short arm of chromosome 16, including ∼20 genes: *PDXDC1* , *NTAN1* , *RRN3* , *NPIPAS* , *MPV17L* , *C16orf45* , *KIAA0430* , *NDE1* (#MIM 609449), *MYH11* (#MIM 160745), *FOPNL* , ABCC6 (#MIM 603234), and ABCC1. The rearranged region has not been reported in the database of the copy number variants (CNVs).

## Discussion

Microtia is a congenital condition characterized by the underdevelopment or absence of the external ear, typically resulting in a smaller or abnormally shaped ear. 12 13 It can occur unilaterally (affecting one ear) or bilaterally (affecting both ears), and its severity can vary widely. 3 The outer ear begins its development during the fifth week of gestation, which is driven by the mesenchyme of the first and second pharyngeal arches on the ventral surface of the embryo. 13 Veugen et al 25 maintained that during the gestation, almost the entire auricle is derived from the second pharyngeal arch with the exception of the tragus and the anterior external auditory meatus. The process of ear formation started at the 5th week of gestation is completed at 12th week. Migration of the outer ear to its normal placement occurs around the 20th week. 18 From the second branchial arch also, stapedial artery arises. 2 The mechanism of the auricular malformation is not well known. Vascular disruption has been suggested as possible causal factor through several ways: occlusion of the artery, vasoconstriction, and underdevelopment of the arterial system. These dysfunctions may cause failure of blood supply causing localized ischemia and tissue necrosis with consequent block of the normal ear development. 11 12 13 Depending on other organ abnormalities, microtia can be classified into nonsyndromic and syndromic types, among which the nonsyndromic types are the most frequent. 26 The syndromic microtia is associated with several pathogenic genes, 5 11 12 13 14 15 16 18 19 20 while nonsyndromic microtia is believed to result from a combination of genetic and environmental factors. According to recent findings, however, some genes are suspected to be involved in the disease process. 26 27 28 Microtia often leads to hearing impairment or complete deafness in the affected ear due to the structural abnormalities of the ear canal and middle ear. Surgical procedures, such as ear reconstruction or the use of hearing aids, are commonly employed to address the functional and cosmetic aspects of microtia. 26 With regard to the treatment of this disorder is a challenge for specialists in this field and there is need to create a collaborative and multidisciplinary net by all of them. As suggested by Truong et al, 29 the list of consultants should include otorhinolaryngology, pediatric audiology, genetics, craniofacial clinic, plastic surgery, oral-maxillofacial surgery, microtia specialist, and speech pathology. 29 For ear malformations as those presented by the proband affected by microtia-associated aural atresia, auricular reconstruction for the microtia must precede the aural atresia surgery. 1 Surgical and nonsurgical options for hearing management and auricular reconstruction, and the treatment timeline for each option need to be carefully considered. 30 The age 3 to 5 years are earliest age suggested for alloplastic porous polyethylene reconstruction. Any ear canal surgery should be done before or currently with alloplastic reconstruction. The age of 5 years is considered the earliest age for an osseointegrated anchor for prosthetic use. Prosthetics may be worn with adhesive prior to the age of 5 years. The age of 5 to 9 years is reported the earliest age for autologous cartilage reconstruction. Any ear canal surgery is ideally done after microtia reconstruction. 29 30

The 16p13.11 microdeletion has been reported with wide clinical expressivity ranging from apparently normal individuals to syndromic cases with severe neurological impairments as congenital severe microcephaly and congenital heart defects. 21 22 23 Clinical presentation may express with facial dysmorphism, developmental speech and language delay, cognitive impairment, schizophrenia, autism spectrum disorder, psychiatric and behavioral problems, and diverse spectrum of sporadic epilepsy syndromes. One of the first reports was described by Ullmann et al, who detected the 1.5 Mb deletion in 16p13.1 in three unrelated patients with ID and other abnormalities. 31 Two years later, Hannes et al. 32 in a screening study of 1027 patients with intellectual disability (ID) and/or multiple congenital anomalies identified five subjects with 1.65 Mb deletion in 16p12. A genome-wide approach was used by Heinzen et al 33 to evaluate the role of 16p13.11. The study was conducted in 3,812 patients with a diverse spectrum of epilepsy syndromes. Large deletions (> 100 Kb) at 16p13.11 were observed in 23 patients, whereas in the control group, no deletions more than 16 Kb were found. Recently, Granata et al 27 reported on three distinct families in which two individuals were affected by inherited deletion in 16p13.11 and 16p13.11p12.3, respectively, and another third had a de novo 16p13.11 microdeletion. All four patients showed variable degrees of ID; two of them were also affected by language delay and dysmorphism, while in a single case was observed cryptorchidism and in another one stereotypes. To note, the stereotypes were found also in our proband.

In the present study, the prominent clinical feature shown by the proband is a severe unilateral microtia with aural atresia, and a linear subcutaneous thickening of fibrotic consistence following partially the course of the mandible, which is not involved. Other clinical manifestations consist of hair white patch in the parietotemporal region, unspecific facial dysmorphism, precocious teething, marked speech delay with a good social approach, hyperactivity, and abnormal stereotypic movements. According to the cytogenetic-molecular analysis, the detected CNV has been associated with the intellectual delay, multiple congenital anomalies, and epilepsy. 32 Second, the role of most deleted genes ( *PDXDC1* , *NTAN1* , *RRN3* , *NPIPA1* , *MPV17L* , *NDE1* , *MYH11* , *ABCC6* , and *ABCC1* ) has been described in a previous finding examining the genotype–phenotype correlation of this susceptible locus. 28 Single or in combination genetic causes have been a relevant role in the onset of this anomaly. The 16p13.11 locus, deleted in our patient, is a genomic hotspot particularly rich in low-copy repeats (LCRs), highly homologous DNA sequences that increase the likelihood of copy number mutation through nonallelic homologous recombination. 27 The 16p13.11 region has been subdivided into three single-copy sequence intervals called interval I, II, and III, each flanked by LCRs. Typical 16p13.11 microdeletions have wide variable size (from 0.8 to 3.3 Mb) and encompass one or more of the three intervals. 27 In the general population, the prevalence of the 16p13.11 microdeletion has been estimated to be around 0.04%. Among the genes mapped in the 16p13.11 locus, *NDE1* , *NTAN1* , and *MYH11* are the main genes responsible of the microtia phenotype. 27 *NDE1* gene is expressed in the brain and its protein is known to act in microtubule organization, mitosis, and neural migration: its dysfunction seems to cause neurodevelopmental anomalies and microcephaly. *NTAN1* is involved in protein degradation and has been associated with behavioral disorders. Ultimately, *MYH11* coding for the major contractile protein in smooth muscle cells causes genetic disorders of the musculature. 27

Ultimately, the wide clinical expression of the 16p13.11 microdeletion that comprises unaffected and affected mutation carriers, of which the latter may feature neurological deficits and congenital anomalies ranging from being mild to very severe is still matter to debate. It may be due to several reasons such as incomplete penetrance, variable expressivity, unmasking of a recessive variant, different size of the microdeletion, genes affected and additional genetic variants outside the deletion, or a combination of these factors. 27

## Conclusion

Auricular malformations are a field of great clinical interest as it involves patients, families, and specialists. Fortunately, great progresses have made in the diagnosis and treatment of this condition. We have reported a new possible association of the 16p13 microdeletion with a patient presenting a severe microtia and aural atresia.

## References

[1] Eavey R D Ear malformations. What a pediatrician can do to assist with auricular reconstruction Pediatr Clin North Am 1996 43 06 1233 1244 8973510 10.1016/s0031-3955(05)70516-2

[2] Andrews J Kopacz A A Hohman M H Ear microtia Treasure Island (FL) StatPearls Publishing 2022Jan-. Available at:https://www.ncbi.nlm.nih.gov/books/NBK563243/33085390

[3] Luquetti D V Leoncini E Mastroiacovo P Microtia-anotia: a global review of prevalence rates Birth Defects Res A Clin Mol Teratol 2011 91 09 813 822 21656661 10.1002/bdra.20836 PMC3405852

[4] Canfield M A Langlois P H Nguyen L M Scheuerle A E Epidemiologic features and clinical subgroups of anotia/microtia in Texas Birth Defects Res A Clin Mol Teratol 2009 85 11 905 913 19760683 10.1002/bdra.20626

[5] Cox T C Camci E D Vora S Luquetti D V Turner E E The genetics of auricular development and malformation: new findings in model systems driving future directions for microtia research Eur J Med Genet 2014 57 08 394 401 24880027 10.1016/j.ejmg.2014.05.003 PMC4143470

[6] Harris J Källén B Robert E The epidemiology of anotia and microtia J Med Genet 1996 33 10 809 813 8933331 10.1136/jmg.33.10.809 PMC1050757

[7] Al-Sulaimani A K Al-Khabori M S Haridi K M Al-Busaidi S S Prevalence and characteristics of microtia in Oman: 37 Years analysis J Plast Reconstr Aesthet Surg 2023 76 292 294 36509652 10.1016/j.bjps.2022.10.047

[8] Ma J Zhou W-H [Genetic characteristics of microtia-associated syndromes in neonates] Zhongguo Dang Dai Er Ke Za Zhi 2022 24 06 614 619 35762425 10.7499/j.issn.1008-8830.2203008 PMC9250400

[9] Estandia-Ortega B Reyna-Fabián M E Velázquez-Aragón J A González-Del Angel A Fernández-Hernández L Alcántara-Ortigoza M A The enigmatic etiology of oculo-auriculo-vertebral spectrum (OAVS): an exploratory gene variant interaction approach in candidate genes Life (Basel) 2022 12 11 1723 36362878 10.3390/life12111723 PMC9693117

[10] Quinn S Kavanagh K McArdle L Betts D Lynch S-A Unilateral microtia found in association with a de-novo 20q13.33 deletion, is there a causal link? Clin Dysmorphol 2023 32 01 39 42 36503924 10.1097/MCD.0000000000000440

[11] Gendron C Schwentker A van Aalst J A Genetic advances in the understanding of microtia J Pediatr Genet 2016 5 04 189 197 27895971 10.1055/s-0036-1592422 PMC5123892

[12] Lu M Lu X Jiang H Pan B Review of preferential suspicious genes in microtia patients through various approaches J Craniofac Surg 2020 31 02 538 541 31977690 10.1097/SCS.0000000000006244

[13] Luquetti D V Heike C L Hing A V Cunningham M L Cox T C Microtia: epidemiology and genetics Am J Med Genet A 2012 158A 01 124 139 22106030 10.1002/ajmg.a.34352 PMC3482263

[14] Kohlhase J SALL1 mutations in Townes-Brocks syndrome and related disorders Hum Mutat 2000 16 06 460 466 11102974 10.1002/1098-1004(200012)16:6<460::AID-HUMU2>3.0.CO;2-4

[15] Celse T Tingaud-Sequeira A Dieterich K *OTX2* duplications: a recurrent cause of oculo-auriculo-vertebral spectrum J Med Genet 2023 60 06 620 626 36368868 10.1136/jmg-2022-108678

[16] Hao S Jin L Li C Mutational analysis of GSC, HOXA2 and PRKRA in 106 Chinese patients with microtia Int J Pediatr Otorhinolaryngol 2017 93 78 82 28109504 10.1016/j.ijporl.2016.12.026

[17] Quiat D Timberlake A T Curran J J Damaging variants in FOXI3 cause microtia and craniofacial microsomia Genet Med 2023 25 01 143 150 36260083 10.1016/j.gim.2022.09.005 PMC9885525

[18] Aguinaga-Ríos M Frías S Arenas-Aranda D J Morán-Barroso V F [Microtia-atresia: clinical, genetic and genomic aspects] Bol Méd Hosp Infant México 2014 71 06 387 395 10.1016/j.bmhimx.2014.11.001 29421636

[19] Bartel-Friedrich S Congenital auricular malformations: description of anomalies and syndromes Facial Plast Surg 2015 31 06 567 580 26667631 10.1055/s-0035-1568139

[20] Veltman J A Jonkers Y Nuijten I Definition of a critical region on chromosome 18 for congenital aural atresia by arrayCGH Am J Hum Genet 2003 72 06 1578 1584 12740760 10.1086/375695 PMC1180319

[21] Tropeano M Andrieux J Collier D A Clinical utility gene card for: 16p13.11 microdeletion syndrome Eur J Hum Genet 2014 22 05 10.1038/ejhg.2013.230 PMC3992581 24105370

[22] Tan L Bi B Zhao P Severe congenital microcephaly with 16p13.11 microdeletion combined with NDE1 mutation, a case report and literature review BMC Med Genet 2017 18 01 141 29191162 10.1186/s12881-017-0501-9 PMC5709987

[23] Smith A E Jnah A Newberry D Chromosome 16p13.11 microdeletion syndrome in a newborn: a case study Neonatal Netw 2018 37 05 303 309 30567812 10.1891/0730-0832.37.5.303

[24] Hunter M Spiller K J Dominique M A Microglial transcriptome analysis in the rNLS8 mouse model of TDP-43 proteinopathy reveals discrete expression profiles associated with neurodegenerative progression and recovery Acta Neuropathol Commun 2021 9 01 140 34412701 10.1186/s40478-021-01239-x PMC8377972

[25] Veugen C CAFM Dikkers F G de Bakker B S The developmental origin of the auricula revisited Laryngoscope 2020 130 10 2467 2474 31825094 10.1002/lary.28456 PMC7540330

[26] Chen X Xu Y Li C Key genes identified in nonsyndromic microtia by the analysis of transcriptomics and proteomics ACS Omega 2022 7 20 16917 16927 35647449 10.1021/acsomega.1c07059 PMC9134388

[27] Granata P Cocciadiferro D Zito A Whole exome sequencing in 16p13.11 microdeletion patients reveals new variants through deductive and systems medicine approaches Front Genet 2022 13 798607 35368691 10.3389/fgene.2022.798607 PMC8965081

[28] Arslan A B Zamani A G Yıldırım M S Novel findings, mini-review and dysmorphological characterization of 16p13.11 microduplication syndrome Int J Dev Neurosci 2022 82 04 289 294 35470466 10.1002/jdn.10188

[29] Truong M T Liu Y-CC Kohn J Integrated microtia and aural atresia management Front Surg 2022 9 944223 36636584 10.3389/fsurg.2022.944223 PMC9831057

[30] Patel K R Benchetrit L Ronner E A Development of an interdisciplinary microtia-atresia care model: a single-center 20-year experience Laryngoscope Investig Otolaryngol 2022 7 06 2103 2111 10.1002/lio2.896 PMC9764815 36544952

[31] Ullmann R Turner G Kirchhoff M Array CGH identifies reciprocal 16p13.1 duplications and deletions that predispose to autism and/or mental retardation Hum Mutat 2007 28 07 674 682 17480035 10.1002/humu.20546

[32] Hannes F D Sharp A J Mefford H C Recurrent reciprocal deletions and duplications of 16p13.11: the deletion is a risk factor for MR/MCA while the duplication may be a rare benign variant J Med Genet 2009 46 04 223 232 18550696 10.1136/jmg.2007.055202 PMC2658752

[33] ATP1A3 Working Group Heinzen E L Arzimanoglou A Brashear A Distinct neurological disorders with ATP1A3 mutations Lancet Neurol 2014 13 05 503 514 24739246 10.1016/S1474-4422(14)70011-0 PMC4238309

